# Multiple Concurrent Illnesses Associated with Anemia in HIV-Infected and HIV-Exposed Uninfected Children Aged 6–59 Months, Hospitalized in Mozambique

**DOI:** 10.4269/ajtmh.19-0424

**Published:** 2020-01-13

**Authors:** Caitlyn Duffy, Darlenne B. Kenga, Tebeb Gebretsadik, Fabião E. Maússe, Alice Manjate, Ernesto Zaqueu, Hermenegilda F. Fernando, Ann F. Green, Jahit Sacarlal, Troy D. Moon

**Affiliations:** 1 Department of Pediatrics, Vanderbilt University Medical Center, Nashville, Tennessee;; 2 Department of Microbiology, Faculty of Medicine, University Eduardo Mondlane, Maputo, Mozambique;; 3 Department of Biostatistics, Vanderbilt University Medical Center, Nashville, Tennessee;; 4 Central Hospital Quelimane, Quelimane, Mozambique;; 5 General Hospital Mavalane, Maputo, Mozambique;; 6 Vanderbilt Institute for Global Health, Vanderbilt University Medical Center, Nashville, Tennessee

## Abstract

Anemia is an increasingly recognized problem in sub-Saharan Africa. To determine the magnitude, severity, and associated factors of anemia among hospitalized children aged 6–59 months, HIV-infected and HIV-exposed uninfected children (a child born to a known HIV-infected mother) with a documented fever or history of fever within the prior 24 hours of hospital admission (*N* = 413) were included in this analysis. Of 413 children enrolled, 364 (88%) were anemic, with 53% classified as mild anemia (hemoglobin [Hb] 7–9.9 g/dL). The most common diagnoses associated with hospital admission included acute respiratory illness (51%), malnutrition (47%), gastroenteritis/diarrhea (25%), malaria (17%), and bacteremia (13%). A diagnosis of malaria was associated with a decrease in Hb by 1.54 g/dL (*P* < 0.001). In HIV-infected patients, malaria was associated with a similar decrease in Hb (1.47 g/dL), whereas a dual diagnosis of bacteremia and malaria was associated with a decrease in Hb of 4.12 g/dL (*P* < 0.001). No difference was seen in Hb for patients on antiretroviral therapy versus those who were not. A diagnosis of bacteremia had a roughly 4-fold increased relative odds of death during hospitalization (adjusted odds ratio = 3.97; 95% CI: 1.61, 9.78; *P* = 0.003). The etiology of anemia in high-burden malaria, HIV, tuberculosis, and poor nutrition countries is multifactorial, and multiple etiologies may be contributing to one’s anemia at any given time. Algorithms used by physician and nonphysician clinicians in Mozambique should incorporate integrated and non–disease specific approaches to pediatric anemia management and should include improved access to blood culture.

## INTRODUCTION

Children are the most vulnerable population to anemia worldwide. From 1993 to 2005, the global prevalence among preschool children was roughly 50%, with approximately 30% of these coming from sub-Saharan Africa.^1–5^ Anemia in early childhood is a leading cause of hospital admission in sub-Saharan Africa and has been associated with reduced cognitive development and growth, and poor immune function, and is responsible for a high proportion of the malaria-related deaths each year.^6–9^ Public health efforts to target anemia have had limited success because of its multifactorial nature and have largely focused on populations considered to be at high risk, namely, pregnant women and children younger than 5 years in low-resource settings.^10–12^ Anemia in sub-Saharan Africa has historically been attributed to three principal causes: malaria, poor nutritional status with micronutrient deficiencies, and helminth infections, with HIV-associated anemia becoming a growing cause of total anemia burden due to opportunistic infections, adverse drug reactions, and untreated immunosuppression.^13,14^

In Mozambique, the contributors to anemia in hospitalized children aged less than 5 years largely mirror what is seen in other sub-Saharan Africa countries, with malnutrition, iron deficiency, malaria, and HIV being reported as the main associated factors.^15^ Mozambique is a country of approximately 29 million people as of 2017 and has one of the highest HIV prevalence rates in the region (13.2% in 2015).^16,17^ The number of persons on antiretroviral therapy (ART) in recent years has increased nearly 4-fold, from 309,000 in 2012 to 1.2 million in 2017.^18^ Mozambique is further classified as one of the 30 high tuberculosis (TB) burden countries, in which it is estimated that nearly 60% of TB patients are coinfected with HIV.^19^ Malnutrition remains a significant problem in the country, with stunting prevalence one of the highest in Africa, roughly 44% in 2015.^20^ Malaria accounted for approximately 30% of all deaths in 2017, with the bulk of this mortality occurring in children aged less than 5 years.^21,22^ Finally, a study of all children aged less than 5 years hospitalized in Maputo in February and March 2009 showed a prevalence of pathogenic intestinal parasites at 16.1%.^23^

In recent years, the burden of anemia has been garnering increasing attention; although targeted, context-specific knowledge of its epidemiology and severity across geographic areas remains limited.^11,15^ Because nationally representative data on anemia are frequently insufficient or unavailable, we often estimate its prevalence based on available subpopulation data and then extrapolate to estimate the overall population-level prevalence.^1^ Despite increased attention in the scientific community at large, at the bedside in sub-Saharan Africa, anemia frequently does not get the attention it deserves, likely resulting from clinicians viewing it as a consequence of other disease processes.^15^ This is highlighted by one report, for example, in which adherence to international guidelines for transfusion was poor in the case of severe anemia, and most patients requiring blood did not get it.^24^ As a result, anemia is likely a much more widespread and under-recognized public health problem in low-resource countries, not only contributing to poor health outcomes in children but also limiting their future socioeconomic potential due to impaired cognitive development.^6,15,25^

Our objective was to describe the magnitude, severity, and associated factors of anemia among HIV-infected and HIV-exposed uninfected hospitalized children aged 6–59 months in two regionally distinct areas of Mozambique. Identifying the magnitude of anemia and its associated factors in this high-risk population could inform future interventions and health practices that aim to reduce the prevalence of anemia in Mozambique.

## MATERIALS AND METHODS

### Study design and population.

**Figure 1.**
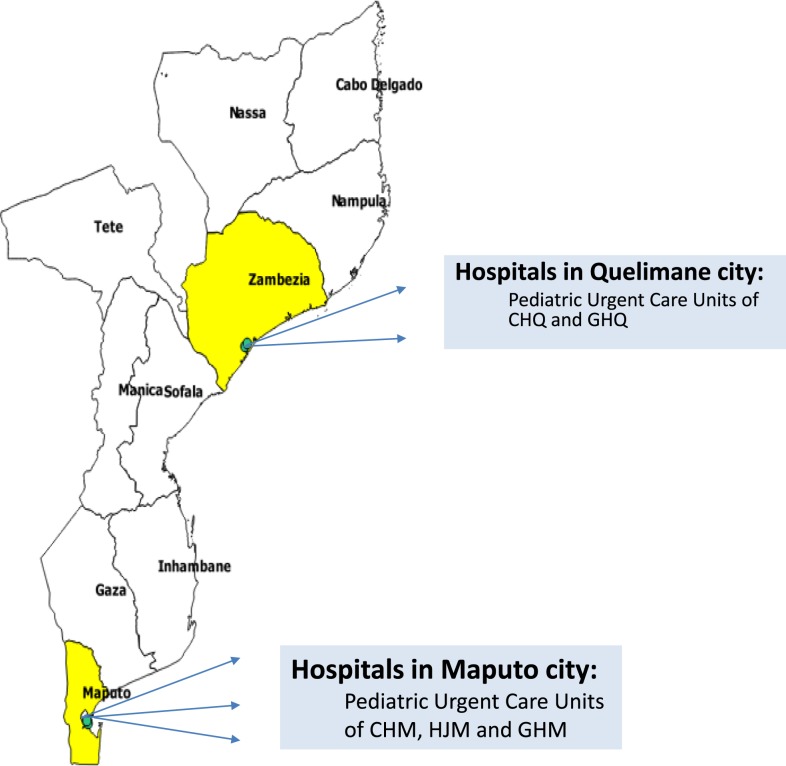
Map of Mozambique with study hospitals identified for Maputo and Quelimane. This figure appears in color at www.ajtmh.org.

We conducted a prospective observational study of HIV-infected and HIV-exposed uninfected children in the cities of Maputo and Quelimane, Mozambique, who were prospectively followed up during their hospital stay. This “parent” study was designed to determine the incidence, etiology, antibiotic sensitivity patterns, and molecular characterizations of culture-confirmed bacteremia in representative rural and urban hospitals in Mozambique. All HIV-infected and HIV-exposed uninfected children aged 0–59 months with a documented axillary temperature of ≥ 37.5°C or rectal temperature ≥ 38.0°C, or a history of fever within the 24 hours before hospitalization between April 1, 2016 and December 31, 2018 were enrolled. Patients were recruited from the pediatric urgent care clinics of three hospitals in Maputo: Central Hospital Maputo, Hospital Jose Macamo, and General Hospital Mavalane; and two hospitals in Quelimane: Central Hospital Quelimane and General Hospital Quelimane. All are tertiary referral hospitals supported by the National Health System (Figure 1).

Using data collected during the course of the parent study, we conducted this anemia analysis on our patient population aged between 6 and 59 months.

### Definitions.

Children were considered HIV positive if they had a documented proof of HIV infection by polymerase chain reaction (PCR) or HIV rapid antibody tests (if ≥ 9 months at time of test), or if they were taking ART in the absence of documented HIV test results. Children admitted with a fever, or a history of fever, and no documented history of HIV, but with documented maternal HIV exposure by self-report, were offered a PCR (if < 9 months old) or an HIV rapid antibody test (if ≥ 9 months old). Those with negative test results were then considered HIV-exposed uninfected. Children admitted with a fever, or a history of fever, and no documented history of HIV and no documented maternal HIV exposure by self-report, although with high clinical suspicion of HIV, were enrolled and offered HIV testing based on the aforementioned. Those with negative test results were considered HIV negative, and there were 18 subjects who were excluded from analysis because of a final designation of being HIV negative or unknown.

For all eligible patients, study staff collected a single blood specimen for bacterial culture before the initiation of antibiotics. Additional diagnostic testing was performed if there was clinical suspicion based on the history or signs/symptoms. Readily available tests included complete blood count, blood chemistries, dipstick urinalysis, chest X-ray, lumbar puncture for chemistries and bacterial culture, stool culture, stool ova and parasites, HIV rapid antibody testing or DNA-PCR, and malaria antigen rapid testing. For patients with a positive malaria rapid test, thick and thin blood films were prepared for confirmation and to quantify *Plasmodium falciparum* parasitemia. There was no available laboratory diagnostics for TB in this age-group.

Severe anemia was defined as a hemoglobin (Hb) concentration of < 7 g/dL, moderate anemia as Hb of 7–9.9 g/dL, mild anemia as Hb of 10–10.9 g/dL, and no anemia if Hb was > 11 g/dL. All patients were followed up until their discharge from the hospital. Possible final disposition included discharged patients, patients who died during hospitalization, or patients who abandoned treatment, meaning they left the hospital against the wishes of the treating clinician.

### Data collection.

Data for the parent study, including this anemia analysis, were collected by study clinicians using a paper-based study instrument and then uploaded into a password protected, tablet-based, online database maintained by the Research Electronic Data Capture consortium (www.project-redcap.org). This allowed for the recording of demographic information, medical and medication history, and information on clinical course while hospitalized. Data quality control was conducted by study investigators who reviewed all completed paper-based study instruments and confirmed the accuracy of data entered into the electronic database.

### Statistical analysis.

Descriptive statistics were used to summarize the participants’ sociodemographic characteristics using frequencies and proportions (for dichotomous or categorical variables) or medians with interquartile ranges for continuous variables. In univariate analysis, we compared factors by anemia status defined as > 11 g/dL, 10–10.9 g/dL, 7–9.9 g/dL, and < 7 g/dL using chi-squared test. In regression analysis, for our response or outcome variable that proxies anemia, we used Hb level (g/dL) without categorization. Using the continuous response of the Hb level maximizes our regression power by using all information levels. We assessed the association of prespecified diagnosed conditions of interest that included bacteremia, malaria, gastroenteritis (GE)/diarrhea, acute respiratory illness (ARI), or malnutrition, with the Hb level as a biomarker response variable using separate regression analyses. Because anemia can be secondary to one’s ART treatment, we also examined the association of ART treatment and Hb level among HIV-infected patients. We examined the interaction effect of concurrent diagnoses using separate regression and a cross-product term (bacteremia and malaria, bacteremia and malnutrition, and malaria and malnutrition) on Hb levels as an exploration analysis. Among our subset of children who were HIV infected, we examined the association of diagnosis with the outcome of in-hospitalization death (yes versus no) using multivariable logistic regression. Each multivariable regression analysis included the child’s age, gender, and health facility as covariates for adjustment.

We had case-wise deletions on ∼4% of subjects because of missing data for infant age (*n* = 18). We performed multiple imputations (MIs) to account for missing age data in multivariable regression as sensitivity analyses.^26^ We present beta coefficient-associating factors with anemia from complete case analysis. Statistical significance was determined using a 2-sided, 5% significance level. Statistical analysis was performed using R version 3.4.0 software (R core team, 2015, R Foundations for Statistical Computing, Viena, Austria, http://www.r-project.org).

### Ethical considerations.

The Mozambican National Bioethics Committee for Health (*Comité Nacional de Bioética para Saúde*) (404/CNBS/14) and the Institutional Review Board of Vanderbilt University Medical Center (IRB#141167) approved this analysis. Informed consent was obtained from the parent or legal guardian of all children enrolled in this study.

## RESULTS

### Patient characteristics.

A total of 413 HIV-infected (95%) and HIV-exposed uninfected (5%) children aged 6–59 months were enrolled in our study between April 1, 2016 and December 31, 2018, of which 57% were male and 36% were aged 6–12 months (Table 1). Two hundred twenty-three children (54%) were recruited from hospitals in Quelimane, and 190 children (46%) from hospitals in Maputo (Figure 1). Three hundred sixty-four (88%) children were anemic at admission, defined by Hb of ≤ 11.0 g/dL. Most (53%) children were classified as having moderate anemia (Hb 7–9.9 g/dL), and the median Hb level was 8.7 g/dL (IQR: 7.3, 10.0). The five most common diagnoses associated with hospital admission included ARI (51%), malnutrition (47%), GE/diarrhea (25%), malaria (17%), and bacteremia (13%). Overall, 40 children (10%) in the cohort died during hospitalization.

**Table 1:** Sociodemographics of children aged 6–59 months hospitalized in Maputo and Quelimane, April 2016–December 2018

*N* = 413	Total, *N* (%)
Gender	
Male	236 (57)
Female	177 (43)
Age (months)	
6–12	148 (36)
13–24	151 (37)
25–59	96 (23)
Missing data	18 (4)
Hospital	
General Hospital Quelimane	128 (31)
Central Hospital Quelimane	95 (23)
General Hospital Mavalane (Maputo)	106 (26)
Hospital Jose Macamo (Maputo)	71 (17)
Central Hospital Maputo	13 (3)
Hb level (g/dL), median [IQR]	8.7 [7.3, 10.0]
Anemia status	
Non-anemic (Hb > 11 g/dL)	49 (12)
Mild anemia (Hb 10–10.9 g/dL)	59 (14)
Moderate anemia (Hb 7–9.9 g/dL)	217 (53)
Severe anemia (Hb < 7 g/dL)	88 (21)
HIV status‡	
Positive	393 (95)
HIV-exposed uninfected	20 (5)
On ART* (*n* = 408)	231 (57)
Hospital diagnosis‡	
ARI	210 (51)
Malnutrition	196 (47)
Gastroenteritis/diarrhea	105 (25)
Malaria†	71 (17)
Bacteremia†	54 (13)
Tuberculosis	25 (6)
Helminth infection	5 (1)
Dermatitis	5 (1)
Seizures	9 (2)
Other	93 (23)
Death during hospitalization	40 (10)

ARI = acute respiratory infection; ART = antiretroviral therapy; Hb = hemoglobin.

* Being on ART refers to HIV-infected children in treatment and HIV-exposed uninfected children receiving ART prophylaxis.

† Laboratory-confirmed diagnosis.

‡ Values could add up to more than 100%.

### Anemia status and diagnosis.

**Figure 2.**
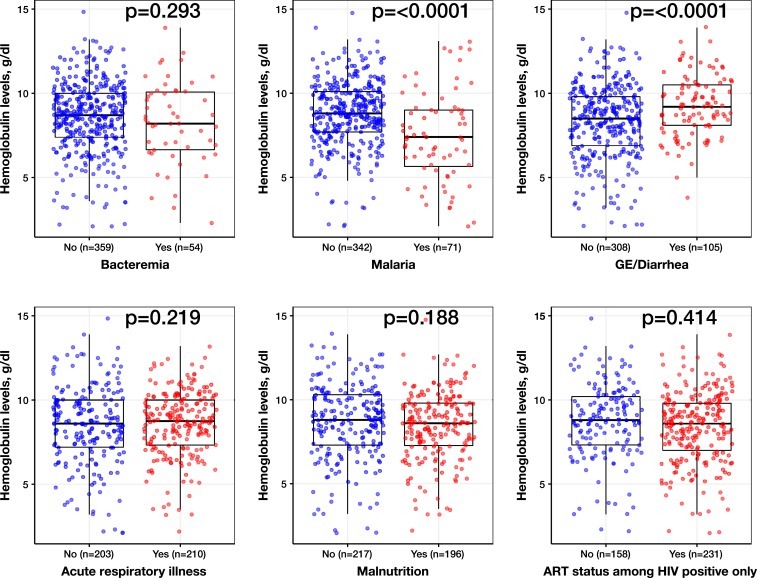
Box plots of hemoglobin level (g/dL) at the time of hospitalization (*y* axis) by the diagnosed conditions of interest (*x* axis). The median (middle bar), first quartile (lower bar), and third quartile (upper bar) are shown. Scatter plot values of each patient’s hemoglobin level was overlaid by diagnosed conditions. The sample size of each group is also shown. *P*-values were obtained from separate multivariable linear regressions that adjusted for child’s age, gender, and health facility/center. *Antiretroviral therapy (ART) status = on ART. This figure appears in color at www.ajtmh.org.

We classified patients by their anemia status as well as the five most common hospital diagnoses encountered (Table 2). Across all diagnoses, the largest proportion of children (between 34% and 59%) was classified as having moderate anemia. Those with a malaria diagnosis had a higher proportion of cases classified as severe anemia (46%, *P* < 0.001), compared with patients without a malaria diagnosis. When we compared distribution of Hb levels by diagnosis, we observed statistically significant lower Hb levels for children diagnosed with malaria (median 7.4 IQR: 5.7–9.0 versus median 8.8 IQR: 7.7–10.1; *P* < 0.0001) and a statistically significant increased Hb level for children diagnosed with GE/diarrhea (*P* < 0.0001) (Figure 2).

**Table 2:** Anemia status (g/dL) of children aged 6–59 months hospitalized in Maputo and Quelimane, Mozambique

*N* = 413	Anemia (g/dL)				
	Non-anemic (Hb > 11), *N* (%)	Mild (Hb 10–10.9), *N* (%)	Moderate (Hb 7–9.9), *N* (%)	Severe (Hb < 7), *N* (%)	*P*-value*
Hospital diagnosis					
Bacteremia (*n* = 54)†	8 (15)	8 (15)	217 (53)	88 (21)	0.25
Malaria (*n* = 71)†	8 (11)	6 (8)	24 (34)	33 (46)	< 0.001
Malnutrition (*n* = 196)	20 (10)	24 (12)	110 (56)	42 (21)	0.41
ARI (*n* = 210)	21 (10)	35 (17)	113 (54)	41 (20)	0.29
GE/diarrhea (*n* = 105)	18 (17)	16 (15)	62 (59)	9 (9)	0.002
On ART (*n* = 231)‡	23 (10)	33 (14)	117 (51)	58 (25)	0.22
Death during hospitalization (*n* = 40)	2 (5)	2 (5)	31 (78)	5 (12)	0.01

ARI = acute respiratory illness; ART = antiretroviral therapy; GE = gastroenteritis; Hb = hemoglobin.

* Overall chi-squared test *P*-value.

† Laboratory-confirmed diagnosis.

‡ Being on ART refers to HIV-infected children in treatment and HIV-exposed uninfected children receiving ART prophylaxis.

We then examined anemia status in children receiving ART. Overall, the largest proportion of children was classified as moderate anemia (51%) (Table 2). No statistically significant difference was detected in the Hb level for patients on ART versus those who were not (Figure 2).

Among both HIV-infected and HIV-exposed uninfected children using separate multivariable linear regression analyses that assessed the association of hospital diagnosis with the Hb level, a diagnosis of malaria was associated with an average decrease in Hb by 1.54 g/dL (*P* < 0.001) versus those who did not have malaria. A diagnosis of GE/diarrhea was associated with an average increase in Hb of 0.92 g/dL (*P* < 0.001) (Table 3). When analyzed among HIV-infected children only, similar trends were seen for both malaria and GE/diarrhea.

**Table 3:** Association of hospital diagnosis and Hb level (g/dL) among HIV-infected and HIV-exposed uninfected children

	HIV-infected and HIV-exposed uninfected children				HIV-infected only	
	Unadjusted β (95% CI)	*P*-value	Adjusted* β (95% CI)	*P*-value	Adjusted* β (95% CI)	*P*-value
Bacteremia†	−0.32 (−0.95, 0.30)	0.313	−0.34 (−0.97, 0.29)	0.293	−0.57 (−1.23, 0.09)	0.092
Malaria†	−1.31 (−1.86, −0.77)	< 0.001	−1.54 (−2.11, −0.96)	< 0.001	−1.47 (−2.06, −0.88)	< 0.001
Malnutrition	−0.21 (−0.64, 0.21)	0.322	−0.31 (−0.78, 0.15)	0.188	−0.35 (−0.83, 0.14)	0.164
Acute respiratory illness	0.11 (−0.31, 0.53)	0.618	0.28 (−0.16, 0.73)	0.219	0.24 (−0.22, 0.70)	0.297
Gastroenteritis/diarrhea	0.90 (0.42, 1.37)	< 0.001	0.92 (0.42, 1.42)	< 0.001	0.92 (0.40, 1.44)	< 0.001

Hb = hemoglobin.

* Multiple linear regression models for hospital diagnosis and Hb levels as a biomarker for anemia.

† Laboratory-confirmed diagnosis.

A large proportion of our HIV-infected population presented with multiple concurrent illnesses at the time of hospitalization. When we explored the interaction effect of these concurrent illnesses on Hb levels, we found that in HIV-infected patients admitted with both a bacteremia and a malaria diagnosis concurrently, there was an average decrease in Hb of 4.12 g/dL compared with children without bacteremia or malaria (*P* < 0.001). We additionally looked at HIV-infected patients with a dual diagnosis of malaria and malnutrition (average Hb decrease of 1.28 g/dL, *P* < 0.0001) and dual diagnosis of bacteremia and malnutrition (average Hb decrease of 0.67 g/dL, *P* = 0.156). Although both saw relative decreases in Hb, the decreases were of similar magnitude to malaria alone or bacteremia alone (Table 4).

**Table 4:** Among HIV-infected children, the association of multiple concurrent hospital diagnoses with hemoglobin level

Hospital diagnosis	Adjusted contrast* β (95% CI)	*P*-value
Bacteremia and malaria (vs. no bacteremia and no malaria)	−4.12 (−5.85, −2.39)	< 0.0001
Bacteremia and malnutrition (vs. no bacteremia and no malnutrition)	−0.67 (−1.65, 0.30)	0.156
Malaria and malnutrition (vs. no malaria and no malnutrition)	−1.28 (−2.26, −0.29)	< 0.0001

* Separate multiple linear regressions were used to assess the interaction of prespecified diagnosis and to estimate the contrasts of interest.

### Hospital mortality.

In multiple logistic regression, HIV-infected patients who presented to the hospital with bacteremia had a roughly 4-fold increased relative odds of dying during their hospitalization (*P* = 0.003). Those with ARI had a slightly higher relative odds of death during their hospitalization (adjusted odds ratio [aOR]: 1.18; 95% CI: 0.29–2.06; *P* = 0.009) (Table 5). There was no statistically significant association with in-hospital death in patients presenting with malaria (aOR: 0.55; 95% CI: 0.14, 2.07; *P* = 0.373).

**Table 5:** Among HIV-infected children, the association of diagnosis with death during hospitalization using multiple logistic regression

	Adjusted odds ratio* (95% CI)	*P*-value
Anemia (per 1 g/dL decrease)	1.02 (0.87, 1.20)	0.767
Bacteremia†	3.97 (1.61, 9.78)	0.003
Malaria†	0.55 (0.14, 2.07)	0.373
Malnutrition	1.88 (0.80, 4.43)	0.147
Acute respiratory illness	1.18 (0.29, 2.06)	0.009
Gastroenteritis/diarrhea	0.95 (0.40, 2.28)	0.915

* Adjusted odds ratio covariates were child gender, age, and health facility where hospitalized.

† Laboratory-confirmed diagnosis.

## DISCUSSION

The majority of patients (88%) in our cohort of children aged 6–59 months were anemic on admission, with 53% being moderately anemic and 21% being severely anemic. This is much higher than the global prevalence of anemia (∼50%) reported for similarly aged children and slightly higher than what has been reported elsewhere in sub-Saharan Africa (60–70%), underscoring the importance of anemia as a severe public health threat in this region of the world.^1,2,9,15,25^

According to Mozambique’s latest national demographic health survey conducted in 2015, the prevalence of anemia in children aged 6–59 months was 64%, with dramatic differences reported between the two provinces represented in this study, 77% for Zambézia Province and 45% for Maputo City.^17^ Although we saw no statistical differences in the prevalence of anemia by geographic region in our hospital-based cohort, the high overall prevalence of anemia seen in our study and the lack of geographic difference could be due to several factors. First, our cohort is made up of children hospitalized with an acute febrile illness and undoubtedly represents a group with a higher frequency of comorbidities. Second, our study focused on HIV-infected and/or HIV-exposed uninfected children for analysis. HIV itself can inherently cause anemia because of depressed hematopoiesis.^27^ In addition, for patients who have initiated ART and prophylaxis for opportunistic infections with drugs such as sulfamethoxazole/trimethoprim (cotrimoxazole), there is a risk of developing drug-induced anemia. In Ethiopia, for example, in pediatric patients who had initiated ART, the type of regimen and the duration on ART were predictors found to be associated with anemia.^28^ However, the association found between anemia and cotrimoxazole prophylaxis has been mixed. Cotrimoxazole may directly impact erythropoiesis, causing a reduction in red cell number, yet simultaneously lead to increased Hb levels because of its beneficial effects at reducing malaria and other infections.^29–31^ Even still, our study results suggest a cause for concern, as HIV-associated anemia has also been shown to increase the risk of mortality.^13^

Concern about the high prevalence of anemia in our cohort is heightened by the fact that many HIV-infected and HIV-exposed uninfected children in Mozambique are at increased risk for other processes which affect the production of red blood cells, such as nutritional deficiencies from chronic malnutrition, chronic blood loss from helminth infections, and endemic infections such as malaria and TB.^13,32–34^ Moreover, children hospitalized in Mozambique frequently have multiple concurrent illnesses that can simultaneously contribute to anemia. We found that children aged 6–59 months who were diagnosed with malaria had statistically significant decreases in Hb levels, with differences in the average Hb level that appears enhanced in those concomitantly diagnosed with both malaria and bacteremia. In addition, HIV-infected children diagnosed with either bacteremia or ARI were at increased odds of death during their hospitalization.

Therefore, anemia observed in regions with a high burden of HIV infection requires more attention and is likely more multifactorial than that observed in HIV-negative populations.^35^ In Mozambique, specific guidelines have been created for the management and treatment of HIV-infected ambulatory adults with anemia to steer clinician approaches away from empiric treatment and more toward an evidence-based approach to diagnosis and treatment. However, similar tools are less specific for hospitalized patients and for pediatric populations.^13^

Anemia management and treatment for children in Mozambique are largely based on the WHO’s Integrated Management of Childhood Illness (IMCI) protocols. These protocols call for the provision of a package of treatments for mild and moderate anemia, which includes iron supplementation, anti-helminth treatment, and oral antimalarials, if a malaria test is positive; or urgent referral to a hospital if the child has severe anemia.^36^ Although this syndromic diagnosis and management approach do rely on strong evidence as to the most common causes of anemia in this age-group, its implementation is frequently applied without attempt to ascertain the true cause or contributors to one’s anemia, nor trying to determine which component of the treatment package subsequently contributed to one’s improvement.

Strategies for the control of anemia call for the implementation of mass prevention efforts that target, once again, “the most common causes of anemia.” Over the last few decades, we have seen significant investments in efforts targeting anemia’s largest contributors, such as campaigns for iron and multivitamin supplementation, deworming campaigns, and campaigns to prevent malaria, including bed net distribution and indoor residual spraying, among others.^12^ In many high HIV burden countries, such as Mozambique, these campaigns have been implemented simultaneously with large-scale increases in access to HIV care and treatment initiatives.^37–39^ Yet, despite all these, the global prevalence of anemia remains unacceptably high with only modest improvements over time.^2,12^

One reason for this may be that all too often, anemia is dealt with through a lens from which it is seen as a secondary consequence of many other processes, each that individually takes priority in control strategy debates. rather than anemia serving as the primary outcome metric from which multiple etiologies are attacked simultaneously for improvement. For example, Mozambique implements IMCI strategies for syndromic management of anemia; they implement broadscale malaria control programs, conduct frequent deworming and vitamin supplementation campaigns, and have regionally targeted acute and chronic malnutrition programs and large national programs for HIV care and treatment; all strategies recommended for the control of anemia. However, Mozambique lacks a harmonized strategy for coverage and uptake of each of these initiatives at the same time and place. This has resulted in incomplete or disjointed coverage, making it difficult to measure the impact of any individual or concurrent program being implemented.

Moreover, other less common yet still significant contributors to anemia are not being sufficiently addressed in Mozambique, as these are typically only included in small pilot programs or for research in a limited population. For example, in our hospitalized cohort, bacteremia contributed to lower Hb concentrations overall and a higher likelihood of death, yet the vast majority of Mozambique’s population is not served by the handful of health facilities that have access to blood culture machines. We believe the limited scale of microbiology capacity in-country and its resulting undetected bacteremia to be an important contributor to the elevated prevalence of anemia in Mozambique and an important cause of preventable morbidity and mortality in Mozambican children with HIV.^40^ In addition, other studies in Mozambique have shown that although not common, disorders of Hb production do exist. For example, α-thalassemia is present in about 50% of patients tested, potentially interfering with strategies to use Hb level as a marker of iron status and lessening justifications for iron supplementation as an anemia control measure.^14,41,42^

This study has several limitations. First, the study’s primary objective was to determine the incidence, etiology, and other characteristics of bloodstream infections in febrile hospitalized children and, as such, was not designed specifically to identify the various etiologies of anemia in this cohort. We had age covariate missing data (4%), and its missingness did not appear related to Hb values. Further sensitivity analysis conducted by applying MI methods had similar results (data not shown) when compared with the main analysis. This said, we cannot completely rule out the impact of age covariate missing or unmeasured confounders as with any observational study. The definitions and diagnostic testing used for determining one’s HIV status are based on Mozambican national protocols; however, it must be recognized that some children older than 9 months with a positive HIV rapid test could represent an HIV-exposed uninfected child with continued presence of maternal HIV antibodies causing the test to be falsely positive. Nonetheless, we feel then likelihood of this to be very low, if any. Being a hospitalized study of HIV-infected and HIV-exposed uninfected children, our findings likely represent the severe end of the spectrum when it comes to anemic children and a higher proportion of concomitant morbidity and mortality. Future studies should be designed and adequately powered specifically to evaluate the effect of anemia on mortality. Finally, our findings cannot be generalized to the general population of children aged 6–59 months in Mozambique, and our regional focus on Zambézia and Maputo Provinces may limit generalizability to other regions of Mozambique as well.

## CONCLUSION

In a hospitalized cohort of HIV-infected and HIV-exposed uninfected children aged 6–59 months, the prevalence of anemia was high, with most categorized as moderate anemia. The etiology of anemia in high burden malaria, HIV, TB, and poor nutrition countries, such as Mozambique, is multifactorial, and multiple etiologies may be contributing to one’s anemia at any given time. It is important to involve the numerous health programs for malaria prevention and treatment, malnutrition, HIV care and treatment, TB control, and helminth control in a more harmonized approach. For example, health authorities could hold strategic planning meetings on childhood anemia, inviting representatives from each of the aforementioned sectors to map out the timing and coverage of interventions to be implemented so that they are integrated and leverage each other for maximum effect. Finally, clinical algorithms used by physician and nonphysician clinicians in Mozambique should incorporate integrated and non–disease-specific approaches to pediatric anemia management and should include improved access to blood culture.
